# Exposure to interparental violence and psychosocial maladjustment in the adult life course: advocacy for early prevention

**DOI:** 10.1136/jech.2008.077750

**Published:** 2009-06-05

**Authors:** C Roustit, E Renahy, G Guernec, S Lesieur, I Parizot, P Chauvin

**Affiliations:** 1 INSERM, UMR S707, Research Group on the Social Determinants of Health and Health Care, Paris, France; 2 UPMC Univ Paris 06, Paris, France; 3 Centre Maurice Halbwachs, Research Group on Social Inequalities (CNRS-EHESS-ENS), Paris, France; 4 AP-HP, Hôpital Saint Antoine, Department of Public Health, Paris, France

## Abstract

**Background:**

Early family-level and social-level stressors are both assumed to be the components of two main path models explaining the association between exposure to interparental violence in childhood and its long-term consequences on mental health explored through life-course epidemiological studies.

**Aims:**

To investigate the association between exposure to interparental violence in childhood and mental health outcomes in adulthood when taking into account early family and social stressors.

**Methods:**

A retrospective French cohort study of 3023 adults representative of the general population in the Paris metropolitan area was conducted in 2005 through at-home, face-to-face interviews. The outcomes measures were current depression and lifetime suicide attempt, intimate partner violence, violence against children and alcohol dependence.

**Results:**

The adults exposed to interparental violence during childhood had a higher risk of psychosocial maladjustment. After adjusting for family- and social-level stressors in childhood, this risk was, respectively, 1.44 (95% CI 1.03 to 2.00) for depression, 3.17 (1.75 to 5.73) for conjugal violence, 4.75 (1.60 to 14.14) for child maltreatment and 1.75 (1.19 to 2.57) for alcohol dependence.

**Conclusions:**

The adult consequences of parental violence in childhood—and this independently of the other forms of domestic violence and the related psychosocial risks—should lead to intensifying the prevention of and screening for this form of maltreatment of children.

Domestic violence, including exposure to interparental violence, has been identified and reported in US surveys over the last three decades. They showed the extent of this societal problem,^1^ which was recognised as a public health burden as regards the consequences of this form of violence on family life and particularly on children. Since the 1990s, children’s exposure to interparental violence has been considered psychologically abusive, and it was specifically listed in the “emotional abuse” category in the US National Center on Child Abuse and Neglect survey.^2^ In Canada, when measured as a form of maltreatment, interparental violence accounted for over a quarter of all substantiated maltreatment reports in 2003.^3^ In France, the first survey estimating the prevalence of violence against women in families was conducted in 2000,^4^ but interparental violence is not yet recognised as a form of child maltreatment. It is only recognised as a risk factor, according to the findings of more recent studies which showed that marital violence and child maltreatment co-occur in families.^5^

Despite different methodological concerns,^6^ ^7^ due to the wide range of experiences that might qualify as exposure to marital violence, the consequences of interparental violence exposure have been largely documented in clinical samples,^8^ such as families in marital therapy, or in specific populations, such as shelter residents.^9^ General population studies are less numerous, but they show that children exposed to interparental violence are at higher risk of negative developmental outcomes and are more likely to exhibit an increased risk of emotional, behavioural, cognitive and social disturbances^10^^–^12 during childhood. Moreover, certain follow-up studies^13^ have shown the long-term adverse effect of exposure to interparental violence on mental health and social functioning in adulthood.

Intimate partner violence has been described in an integrated, ecological framework where personal, situational and sociocultural factors interact.^14^ In such a framework, pathways from interparental violence to adverse outcomes in adulthood have been elucidated by two main conceptual models. One of them emphasises family stressors as triggers that determine the developmental pathway from interparental violence during childhood to adverse psychosocial outcomes in adulthood. Family stressors include both the components of the personality of the parent involved in violence processes^15^ and the family/life events closely linked with the occurrence of parental violence, such as family break-ups. The other model emphasises social stressors, such as poverty, unemployment and housing problems, as the determinants of both interparental violence and poor psychosocial functioning in adulthood.^16^ From a psychological perspective, individual characteristics, such as temperament,^17^ can also have a mediating effect on the association between interparental domestic violence and later negative mental health outcomes. Age and gender have a moderating effect, and the earlier the exposure to violence, the greater the problems.^18^ The expression of these problems differs according to gender: girls present with more internalising disorders, boys with more externalising disorders.

The main limitations of the studies conducted in this research field are the lack of large-scale longitudinal datasets including relevant social factors. Moreover, very little is known about how interparental violence combines with other adversities in childhood to promote adult psychosocial maladjustment.

The aim of our study was to investigate the association between interparental violence exposure in childhood and mental health outcomes in adulthood when simultaneously taking into account other family and social stressors in childhood. Our hypothesis was that this adverse early life event has a negative impact on different mental health outcomes throughout the individual’s lifetime, over and above both coexisting familial and social stressors during childhood.

## METHODS

### Study design and setting

The SIRS (Health, Inequalities and Social Ruptures) cohort study is a longitudinal epidemiological survey of the general adult population of the Paris metropolitan area, conducted since 2005 by the National Institute of Health and Medical Research (INSERM). This cohort study was approved by the French authority that protects privacy and personal data (Commission Nationale de l’Informatique et des Libertés; CNIL).

This survey, which has been described elsewhere,^19^ was based on a three-stage cluster random sample of 4560 adults (areas, households, adults) stratified according to the socioeconomic status of the neighbourhoods. We first used the Surveyselect procedure (SAS, version 9.1) to randomly select 50 neighbourhoods (over-representing the poorer neighbourhoods). We then randomly selected households in each neighbourhood. Lastly, we selected the interviewees by the birthday method. Data were collected through at-home, face-to-face interviews during the first wave of data collection between September and December 2005.

### Participants

Of the 4560 selected adults, 28.6% refused to participate, and 3.1% who did not speak French and 1.8% who were too sick or unable to answer the survey were excluded from the study population. Overall, 3023 subjects were included in the cohort, for a participation rate of 71.4%. No individual data were collected from the non-respondents, but according to the four-level socioeconomic categorisation of neighbourhoods used for sample stratification, the response rates were higher in both extreme categories, ie, the richest and poorest neighbourhoods (73.9% and 76.5% respectively), than in the two intermediate categories (70.4% and 69.9% respectively; p = 0.003).

### Data sources and variables

We used the retrospective data on childhood circumstances and the cross-sectional data on adulthood indicators collected at that time.

#### Definition of exposure to interparental violence during childhood and indicators of mental health outcomes in adulthood

Witnessing interparental violence in childhood before the age of 18 years was our main independent variable. It was defined according to the answer to the following question: “Did you witness interparental violence before the age of 18?” (yes/no). Five mental health outcomes were studied: depression, lifetime suicide attempt, two violent intrafamilial conduct disorders and alcohol dependence. Depression was investigated by the Mini-International Neuropsychiatric Interview. The depression index was evaluated by a seven-item scale for measuring the occurrence of depressive symptoms during the last 15 days. The scale’s internal and external validity has been demonstrated in the French population by Lecrubier *et al*.^20^ A binary variable indicated the absence or presence of depression. Attempted suicide was defined as at least one act committed with the intention of taking one’s life over the adult’s lifetime from age 18 years to his/her present age. Suicidal thoughts and the therapeutic management of suicide attempts were not taken into account. Violent intrafamilial conduct disorders in adulthood were identified by questions on lifetime experiences of two indicators: intimate partner violence and perpetrating child maltreatment. These questions were, respectively, the following: “Have you ever engaged in violent behaviour toward your spouse?” (yes, presently/yes, in the past/no); “Have you ever engaged in violent behaviour toward your children?” (yes, presently/yes, in the past/no). Each indicator of conduct disorder was coded according to a two-point outcome variable (0 =  no, 1 =  yes, presently, or yes, in the past). Alcohol dependence was identified by a screening test for alcohol problems, namely, the CAGE questionnaire.^21^ In line with previous studies,^22^ we chose a score of 2 or more to identify drinkers at risk of dependence.

#### Family-level stressors and social-level stressors in childhood and other adjustment variables

Family-level stressors included parent–child relationships and childhood adversities. The perceived quality of parent–child relationships was assessed separately for both the mother and the father by the following question: “Before the age of 18, how would you rate your relationship with (1) your father and (2) your mother?”, reported and then dichotomised as very good or good vs poor or very poor. Childhood adversities before the age of 18 were the following: parental separation or divorce, parental death, parental incarceration, the occurrence of a parental suicide attempt, parental alcoholism and other forms of domestic violence, with respect to which the respondents were asked if they had been physically abused and/or sexually abused before the age of 18. The questions used to assess these characteristics were standard questions about childhood living arrangements used in previous French sociological and health surveys. Social-level stressors during childhood were poor parental health status, housing problems, prolonged parental unemployment and family financial problems during childhood.

In addition to age and gender, we adjusted for socioeconomic factors in adulthood recorded at the time of the survey. Individual socioeconomic status (SES) was assessed on the basis of the following variables: level of education, monthly household income per consumer unit and occupational status (according to the French census nomenclature of professions and social categories).

### Statistical analysis

We investigated the association between exposure to interparental violence during childhood prior to age 18 and psychosocial adjustment outcomes in adulthood. The first step in the analysis consisted of examining the past social and family characteristics of the adults who had been exposed to interparental violence during childhood compared with those who had not, and in analysing the bivariate association between having witnessed interparental violence and mental health outcomes in adulthood by age group. Second, we used a binary logistic regression model for each of these outcomes (baseline model) while controlling for age, gender and adult SES. Third, we introduced in the baseline model: (1) the family-level stressors after adjusting for child sexual abuse and/or physical maltreatment; and (2) the social-level stressors in childhood. Interactions between exposure to interparental violence and significant predictive variables in the final model were systematically tested. The data were weighted in order to take into account the sampling design (which combined multistage random clustering and oversampling of poor neighbourhoods during stratification) and then the post-stratification adjustment according to the general population census data. SPSS 15.0 procedures (SPSS Inc., Chicago, IL, USA) were used to perform the statistical analysis.

## RESULTS

### Characteristics of the study population

The study population included 3023 subjects (53% women and 47% men), 29 of whom (ie, less than 1%) did not provide information about past parental violence. Among the respondents, 16% reported having witnessed interparental violence before the age of 18. This prevalence decreased with subject age, from 18.8% in the 18–36 age group to 13.5% in the 55 and over age group. There were neither gender- or nationality-related differences in interparental violence exposure, except in the oldest age group, where French people reported more exposure to interparental violence than did foreigners. The current SES was the same in both groups, except for the level of education. The respondents with the lowest level of education were more likely to have witnessed interparental violence than those with an intermediate or the highest level of education.

### Association between interparental violence and other adverse childhood experiences

Table 1 shows that having witnessed interparental violence in childhood was significantly associated with numerous family-level stressors, with much higher frequencies (at least twice as high and up to eight times higher for parental alcoholism) of reported poor parent–child relationships; adverse parental life events, such as parental separation or divorce, incarceration, suicide attempt or alcoholism (but not parental death); and physical or sexual abuse among the people who reported having witnessed interparental violence during childhood than among those who did not. Witnessing such violence was also more frequently reported in families with financial problems, serious parental diseases, housing problems or unemployment. Interestingly, these associations were observed and were significant in all the age groups in the study population (except for parental death).

**Table 1:** Comparison, by age group, of the characteristics of the adults who witnessed and did not witness interparental violence in childhood, SIRS cohort, Paris metropolitan area, 2005

	Witnessing of interparental violence in childhood							
	Overall		Age 18–36		Age 37–54		Age >54	
	Yes	No	Yes	No	Yes	No	Yes	No
	(511)	(2483)	(189)	(802)	(185)	(812)	(137)	(869)
*Demographics*								
Age (mean)	42.6	45.7	28.2	27.7	44.3	45.4	67.0	68.7
Female	55.3	52.4	52.0	51.6	54.9	49.6	61.7	56.5
French nationality	88.2	85.9	83.3	83.5	88.5	84.2	97.2*	90.7
*Psychosocial outcomes in adulthood*								
Internalising disorders								
Current depression	18.3***	10.4	18.3***	9.4	19.5**	10.4	16.0	11.7
Lifetime suicide attempt	6.0***	2.3	4.0**	1.2	6.3*	2.7	9.3**	3.2
Intrafamilial conduct disorders								
Intimate partner violence	8.1***	1.9	7.4***	1.1	12.5***	2.5	2.9	2.2
Perpetration of child maltreatment	3.8***	0.6	0	0	7.4***	0.7	0	0.8
Alcohol dependence	17.8***	9.2	16.8*	9.4	21.2**	12.2	14.4**	5.7
*Childhood family-level stressors*								
Poor mother–child relationship	25.8***	10.7	21.4***	9.0	27.6***	11.8	30.8***	11.6
Poor father–child relationship	50.1***	19.0	52.0***	20.1	52.3***	19.3	43.4***	17.4
Parental separation or divorce	32.3***	9.0	43.6***	14.6	24.7***	6.6	23.4***	4.6
Parental death	5.4***	11.2	5.0	7.2	5.2	9.6	6.6**	18.0
Parental incarceration	2.9***	0.6	3.0**	0.6	1.2*	0.1	4.7**	1.1
Parental suicide attempt	7.1***	0.9	7.0***	1.4	7.6***	1.1	6.5***	0.1
Parental alcoholism	26.8***	3.5	26.9***	3.1	28.7***	4.4	23.6***	2.9
Physical abuse in childhood	12.2***	1.5	9.5***	1.2	16.5***	1.7	9.4***	1.9
Sexual abuse in childhood	6.9***	1.5	5.9***	1.7	8.7***	1.6	5.6**	1.2
*Childhood social-level stressors*								
Family financial problems	33.0***	15.2	31.0***	12.5	29.2***	14.6	41.9***	19.3
Poor parental health status	30.0***	19.1	29.4***	17.3	29.9**	18.7	32.1*	21.7
Housing problems	11.1***	4.5	9.5***	3.6	11.1***	2.7	14.2*	7.8
Parental unemployment	16.3***	6.2	21.9***	10.9	12.7***	3.6	10.5***	3.3

*p Value <0.05; **p value <0.01; ***p value <0.001.

### Association between exposure to interparental violence and mental health outcomes

Exposure to interparental violence during childhood was significantly associated with all five adulthood mental health outcomes that were studied, with higher odds ratios for intimate partner violence and perpetrating child maltreatment, but also a significant association with depression, lifetime suicide attempt and alcohol dependence (table 2, baseline model adjusted for age, gender, nationality and SES in adulthood).

**Table 2:** Association between exposure to interparental violence in childhood and five psychosocial maladjustment outcomes in adulthood, SIRS cohort, Paris metropolitan area, 2005

	Internalising disorders		Intrafamilial conduct disorders		Alcohol dependence
	Depression	Lifetime suicide attempts	Intimate partner violence	Perpetrating child maltreatment	(n = 3002)
	(n = 3011)	(n = 3007)	(n = 2668)	(n = 1935)	
	aOR (95% CI)	aOR (95% CI)	aOR (95% CI)	aOR (95% CI)	aOR (95% CI)
*Model 1. Baseline model*					
Witnessed interparental violence in childhood	2.01*** (1.53 to 2.65)	2.80*** (1.75 to 4.48)	4.67*** (2.93 to 7.45)	6.66*** (2.74 to 16.18)	2.24*** (1.62 to 3.09)
*Model 2. Baseline model + childhood family-level stressors*					
Witnessed interparental violence in childhood	1.57** (1.14 to 2.17)	1.29 (0.72 to 2.33)	3.55*** (2.00 to 6.33)	4.81** (1.68 to 13.79)	1.75** (1.19 to 2.57)
Poor mother–child relationship	1.45* (1.05 to 2.00)	2.04** (1.20 to 3.45)	1.85* (1.02 to 3.34)	0.80 (0.25 to 2.55)	0.91 (0.59 to 1.40)
Poor father–child relationship	1.50** (1.13 to 1.98)	1.98** (1.19 to 3.30)	1.13 (0.65 to 1.97)	2.08 (0.74 to 5.88)	1.55* (1.11 to 2.16)
Parental separation or divorce	0.75 (0.52 to 1.07)	0.97 (0.51 to 1.83)	0.82 (0.42 to 1.62)	0.61 (0.14 to 2.62)	0.97 (0.64 to 1.49)
Parental incarceration	1.61 (0.59 to 4.41)	0.36 (0.02 to 5.86)	1.73 (0.36 to 8.22)	NA	0.23 (0.03 to 2.06)
Parental suicide attempt	1.60 (0.80 to 3.21)	2.55 (0.96 to 6.78)	1.66 (0.56 to 4.88)	NA	0.93 (0.36 to 2.42)
Parental alcohol abuse	0.97 (0.62 to 1.49)	1.86 (0.94 to 3.68)	0.58 (0.27 to 1.25)	0.68 (0.16 to 2.92)	1.64 (0.99 to 2.72)
Physical maltreatment in childhood	1.38 (0.80 to 2.35)	1.51 (0.66 to 3.46)	5.62*** (2.79 to 11.31)	7.24** (1.75 to 30.06)	1.06 (0.50 to 2.23)
Sexual abuse in childhood	2.03* (1.11 to 3.74)	2.59* (1.03 to 6.49)	2.57* (1.07 to 6.16)	2.59 (0.55 to 12.22)	1.90 (0.81 to 4.46)
*Model 3. Baseline model + childhood social-level stressors*					
Witnessed interparental violence in childhood	1.75*** (1.31 to 2.34)	2.74*** (1.68 to 4.50)	4.25*** (2.60 to 6.97)	6.77*** (2.67 to 17.14)	2.19*** (1.57 to 3.05)
Family financial problems	1.38* (1.03 to 1.86)	0.62 (0.33 to 1.18)	1.51 (0.87 to 2.61)	1.29 (0.41 to 4.08)	1.12 (0.76 to 1.66)
Poor parental health status	1.05 (0.79 to 1.39)	1.27 (0.76 to 2.11)	1.08 (0.62 to 1.86)	1.76 (0.67 to 4.61)	1.12 (0.80 to 1.56)
Housing problems	1.28 (0.82 to 2.02)	2.51* (1.18 to 5.32)	1.34 (0.61 to 2.95)	2.28 (0.52 to 10.02)	1.35 (0.75 to 2.44)
Parental unemployment	1.53* (1.04 to 2.23)	0.99 (0.45 to 2.19)	1.01 (0.47 to 2.16)	0.24 (0.02 to 2.58)	0.96 (0.55 to 1.66)
*Full model: baseline model + childhood family-level stressors + social-level stressors*					
Witnessed interparental violence in childhood	1.44* (1.03 to 2.00)	1.36 (0.75 to 2.48)	3.17*** (1.75 to 5.73)	4.75** (1.60 to 14.14)	1.75** (1.19 to 2.57)
Poor mother–child relationship	1.49* (1.08 to 2.06)	2.09** (1.23 to 3.56)	2.04* (1.13 to 3.70)	0.84 (0.26 to 2.71)	0.91 (0.59 to 1.40)
Poor father–child relationship	1.43* (1.08 to 1.90)	2.02** (1.20 to 3.38)	1.05 (0.60 to 1.86)	1.84 (0.64 to 5.36)	1.53* (1.09 to 2.15)
Parental separation or divorce	0.71 (0.49 to 1.03)	0.99 (0.52 to 1.88)	0.74 (0.37 to 1.49)	0.62 (0.14 to 2.72)	0.96 (0.62 to 1.47)
Parental incarceration	1.40 (0.50 to 3.89)	0.33 (0.02 to 5.53)	1.57 (0.33 to 7.38)	NA	0.21 (0.02 to 1.96)
Parental suicide attempt	1.49 (0.73 to 3.04)	2.63 (0.97 to 7.16)	1.50 (0.49 to 4.57)	NA	0.90 (0.34 to 2.37)
Parental alcohol abuse	0.98 (0.63 to 1.52)	2.02* (1.01 to 4.04)	0.64 (0.29 to 1.37)	0.76 (0.18 to 3.26)	1.66* (1.00 to 2.76)
Physical maltreatment in childhood	1.34 (0.77 to 2.34)	1.39 (0.60 to 3.18)	6.09*** (2.95 to 12.55)	8.48** (1.99 to 36.20)	1.09 (0.52 to 2.30)
Sexual abuse in childhood	2.02* (1.09 to 3.73)	2.64* (1.06 to 6.62)	2.56* (1.06 to 6.22)	3.01 (0.63 to 14.39)	1.88 (0.80 to 4.42)
Family financial problems	1.32 (0.98–1.80)	0.50 (0.25 to 1.01)	1.59 (0.89 to 2.85)	1.34 (0.40 to 4.55)	1.06 (0.70 to 1.59)
Poor parental health status	0.97 (0.72 to 1.30)	1.09 (0.64 to 1.88)	0.91 (0.51 to 1.62)	1.51 (0.54 to 4.16)	1.04 (0.73 to 1.47)
Housing problems	1.23 (0.76 to 1.97)	2.17 (0.92 to 5.10)	1.19 (0.50 to 2.83)	2.74 (0.57 to 13.13)	1.09 (0.58 to 2.05)
Parental unemployment	1.55* (1.05 to 2.29)	0.96 (0.40 to 2.29)	1.23 (0.55 to 2.71)	0.26 (0.02 to 3.21)	1.09 (0.62 to 1.91)

Comparison of different multivariate logistic regression models: (1) baseline model (adjusted for age, gender, nationality and SES in adulthood); (2) baseline model adjusted for family-level stressors in childhood; (3) baseline model adjusted for social-level stressors in childhood; and (4) baseline model adjusted for both types of stressors in childhood^a^.

*p Value <0.05; **p value <0.01; ***p value <0.001; NA, not applicable.

When childhood family-level stressors were introduced into the model, the effect of having witnessed interparental violence decreased but was still significant, except for lifetime suicide attempts, where the association was significantly lower and no longer statistically significant (table 2, model 2). When childhood social-level stressors were introduced (alone) into the baseline model, the effect of having witnessed interparental violence persisted with similar strengths as in the baseline model (it was even slightly higher for perpetrating child maltreatment; table 2, model 3).

Introducing both family-level and social-level stressors in childhood into the model led to a decrease in the effect of having witnessed interparental violence, but it still persisted significantly for all of our outcomes, except lifetime suicide attempts (table 2, full model). None of the interactions between exposure to interparental violence and significant predictive variables in the final model was significant.

### Gender-related differences

By gender group (results not shown), the risk of depression was higher in women exposed to interparental violence in childhood. On the other hand, the men had a 15-fold higher risk of committing violent acts against their children, women a two-fold higher risk. But intimate partner violence was the same for both genders. Finally, the risk of alcohol dependence was higher in the male respondents from high-conflict families and/or with a parental history of alcoholism, and it was also higher after adjusting for depression. This risk was not observed in the women.

## DISCUSSION

Interparental violence is now widely documented as an adverse childhood experience^23^ and a risk factor for negative health outcomes. Our population-based survey confirms such an association in the entire adult lifetime. We found that exposure to parental violence is associated with depression, suicide attempts, intrafamilial conduct disorders and alcohol dependence in the adult lifetime. We also found that family-level stressors, such as poor parent–child relationships before the age of 18 and parental psychopathologies, were mediating factors only on the association between such experiences and suicide attempts in adulthood. For all the other outcomes, there was an association with interparental violence, despite the adjustment for all the selected family-level stressors and social-level stressors in childhood.

However, the limitations of our study warrant some consideration. First, the marital discord variable was not quantified by means of a standardised scale. Nonetheless, the odds ratios were strong enough to suggest that even mildly violent acts, such as verbal rather than physical acts, have negative consequences in adulthood. In the present study, our goal was not to determine a prevalence rate for this event in the general population, but rather to assess the adulthood consequences of a climate of parental violence perceived by the respondents when they were children. Second, with regard to potential non-response bias in such a population-based cohort study, we indicated above that the response rates were higher in the neighbourhoods in both extreme social categories. If one assumes that negative early life events are more frequent in families living in the poorest neighbourhoods, such differences in response rates would not have biased our estimates. On the other hand, we cannot ascertain the number of subjects who did not respond for reasons associated with parental violence during childhood (subjects who had committed suicide, institutionalised subjects, etc.). Third, retrospective reports of adverse childhood experiences could be subject to recall bias. In the literature, the analysis of the validity of retrospective studies has shown that the prevalence of past events is underestimated.^24^ But adverse childhood experiences can also be recalled to a greater degree by psychologically impaired individuals^25^ as the result of internal biographical coherence. In particular, recalling childhood sexual abuse or maltreatment upon a single question may have been connoted by the subject’s emotional state during the face-to-face interview, which, in the more psychologically fragile subjects, would have induced same-source bias. However, we note that, during the face-to-face interviews, data on these childhood characteristics were collected before the (adult) outcomes variables, which reduces at least the prevarication bias. Lastly, our statistical models were not adjusted for lifetime comorbidities.

We found that adults who had witnessed interparental violence during childhood had had numerous other adverse familial and social experiences and had a higher risk of negative mental health outcomes in adulthood. These associations could be explained by the emotional security hypothesis developed by Davies and Cummings.^26^ Children exposed to parental conflict usually live in dysfunctional families, where their feelings of physical and psychological well-being are threatened. The resulting emotional insecurity, defined as emotional ill-being and emotional regulation difficulties in the face of stress, could constitute a developmental pathway between domestic violence and later psychosocial maladjustment.

Numerous types of adverse childhood experiences, such as parental divorce,^27^ sexual abuse,^28^ physical abuse^29^ and/or socioeconomic disadvantages,^30^ are associated with a higher risk of depression in adulthood. However, the specific impact of exposure to interparental violence on mood disorders has been little studied from early to late adulthood. Previous works^31^ suggested that parental conflict is a strong predictive factor for internalising disorders in adolescents. Our findings, which show that it is also a predictive factor for depression at different ages in adulthood, concur with the notion of life-course continuity in psychopathological disorders.^32^ Adolescent-onset depression, even at the subthreshold level, is associated with a high risk of recurrence in young adulthood,^33^ and with other negative outcomes, such as anxiety, conduct disorders and psychoactive substance use.^34^ Such lifetime comorbidity could explain some of our results, in that exposure to interparental violence was associated with depression but also with violent family conduct disorders and alcohol dependence during adulthood.

Our second indicator of psychosocial functioning was intrafamilial conduct disorders. The strength of the association between parental discord and both child and intimate partner violence partially concurs with the notion of intergenerational transmission of violence. Indeed, physical abuse and/or sexual abuse during childhood were independently associated, respectively, with both outcomes and with child maltreatment. This intergenerational cycle of maltreatment and violence has been explained by different theoretical approaches, most of which are based on a general family of cognitive/behavioural paradigms.^21^ According to the social learning theory, observational learning constitutes the primary process through which children learn maladaptive ways of dealing with conflict and orient their patterns of behaviour. According to the family system theory, family dysfunction affects parenting practices, which, in turn, affects the child’s adjustment. Rooted in the social–cognitive approaches, the cognitive–contextual framework argues for the importance of children’s cognitive processes in shaping the impact of parental conflict on psychosocial adjustment.^35^ Finally, several investigations highlight comorbidity between alcohol abuse and other mental health problems, such as drug use and antisocial behaviour.^36^ In our study, however, alcohol dependence was not a confounding factor in the association between domestic violence in childhood and such violence later in adulthood. This association has been described in other works^37^ and could be explained by different pathways. Multiple traumatic experiences occurring in dysfunctional homes could have a cumulative effect on alcohol use. These negative experiences can lead to post-traumatic stress disorders or anxious states later in life. Furthermore, children from alcoholic and violent parents are more prone to alcohol abuse in adulthood through genetic transmission or the integration of familial social norms.

Our study sought to demonstrate the potential harm of interparental violence to children for their future adulthood life, and this independently of other forms of domestic violence and related psychosocial risks. Such a demonstration warrants further research studies so that these consequences become a cornerstone of any risk–benefit analysis of screening interventions towards intimate partner violence.

**What is already known on this subject**

- Children’s exposure to interparental violence is a form of maltreatment and has consequences on child’s development.
- Early family-level and social-level stressors are both assumed to be the components of two main hypotheses explaining the association between exposure to interparental violence and negative health outcomes in children and later in adulthood.

**What this study adds**

- The adults exposed to interparental violence in childhood have a higher risk of mental health disorders in adulthood than those not exposed, and even after adjusting for other forms of domestic violence.
- Family- and social-level stressors in childhood do not explain this association.
- Intensification of prevention of and screening for domestic violence including interparental violence is a public health issue for the well-being of future generations.
